# Sensor Fault Diagnosis Method Based on *α*-Grey Wolf Optimization-Support Vector Machine

**DOI:** 10.1155/2021/1956394

**Published:** 2021-09-10

**Authors:** Xuezhen Cheng, Dafei Wang, Chuannuo Xu, Jiming Li

**Affiliations:** College of Electrical and Automation Engineering., Shandong University of Science and Technology, No. 579 Qianwangang Road, Qingdao 266590, China

## Abstract

Aimed to address the low diagnostic accuracy caused by the similar data distribution of sensor partial faults, a sensor fault diagnosis method is proposed on the basis of *α* Grey Wolf Optimization Support Vector Machine (*α*-GWO-SVM) in this paper. Firstly, a fusion with Kernel Principal Component Analysis (KPCA) and time-domain parameters is performed to carry out the feature extraction and dimensionality reduction for fault data. Then, an improved Grey Wolf Optimization (GWO) algorithm is applied to enhance its global search capability while speeding up the convergence, for the purpose of further optimizing the parameters of SVM. Finally, the experimental results are obtained to suggest that the proposed method performs better in optimization than the other intelligent diagnosis algorithms based on SVM, which improves the accuracy of fault diagnosis effectively.

## 1. Introduction

The sensor functions as a major detection device in the monitoring system [1–3], the detection accuracy of which will be significantly reduced by breakdown. Additionally, it will affect the performance of the monitoring system and even result in economic losses and casualties in some extreme cases. Therefore, it is necessary to make an accurate diagnosis of sensor faults for ensuring that the monitoring system can operate smoothly and reliably.

When the fault intensity stays low, there would be some forms of sensor failure showing similar characteristics of data distribution, which is a leading cause for the low levels of diagnostic accuracy [4]. In traditional approaches to fault diagnosis [5–7], model-based methods require the establishment of an accurately mathematical model for the research object. In practice, however, it is usually difficult to construct the nonlinear system for mathematical models. With regard to knowledge-based methods, they rely heavily on expert experience, which makes them lack adaptability when new problems arise. Additionally, data-driven methods require the learning of historical data, rather than the exact mathematical models or expert knowledge.

With the rapid advancement of artificial intelligence (AI) technology, the AI-based diagnostic methods have attracted much interest for research in the field of fault diagnosis. In [8], a Recurrent Neural Network (RNN) is put forward to model nonlinear systems, thus achieving fault detection and the isolation of sensors. A very random tree method was proposed to detect and diagnose the faults in sensor networks in [9], which demonstrated strong robustness for processing the signal noise but ignored the fault diagnosis for sensor nodes. In [4], a hybrid continuous density HMM-based ensemble neural networks method is applied to detect and classify sensor node faults.

However, due to the similar distribution of some fault data, it is necessary to train a variety of classifiers for the accurate classification of different faults. Furthermore, a fault diagnosis method intended for chiller sensors is presented in [10], which not only achieves feature extraction by clustering the fault data but also identifies the fault types by setting the clustering indicators.

Abnormal data are considered the most effective indicators of sensor failure, which are nonlinear and enormous and make the data-driven intelligent diagnosis method more suitable for the diagnosis of sensor fault [11–13]. Machine learning algorithm is a commonly used method for intelligent diagnosis, including Neural Networks (NNs), Support Vector Machines (SVMs), and so on. However, the amount of fault samples is usually limited, which leads to a poor manifestation for NN. SVM has attracted much attention due to its capability of dealing with nonlinear and small sample size in fault diagnosis [14, 15], but the correct hyperparameters must be chosen for improved performance. Mechanism of different algorithms may be disparate, and the optimization of key parameters can often improve the performance of the algorithm [16, 17]. Researchers have proposed or improved algorithms to solve optimization problems [18–20] and achieved remarkable results, which gives us some inspiration to choose the appropriate hyperparameters of SVM. Besides, it is an effective strategy to improve the accuracy of diagnosis by adopting an appropriate method for extracting the feature of fault data. However, conventional data feature extraction methods such as Principal Component Analysis (PCA) [21] are more suitable for processing linear data. Also, time-domain parameters can also be taken as the reference indicators for diagnosis, but not all of them are sensitive to all sorts of failure [22].

In order to solve the aforementioned problems, there are a number of solutions proposed in this paper. Firstly, multiple time-domain parameters are extracted from sensor fault data, and the Kernel Principal Component Analysis (KPCA) is conducted to perform Principal Component Analysis of the time-domain parameters. Then, some of the time-domain parameters are refused to obtain the fusion features that can accurately reflect the characteristics of fault. Secondly, an *α* Grey Wolf Optimization (*α*-GWO) arithmetic is proposed to achieve parameter majorization for SVM. The competition mechanism is introduced to enhance the ability of algorithm to conduct search. In the meantime, the dominant position of *α* wolf is reinforced to speed up convergence in the later stage of this algorithm. Finally, the samples comprised of the fusion features are inputted into different diagnostic models for the purpose of training and testing. The experimental results are comparatively analyzed to validate the method proposed in this paper for sensor fault diagnosis.

This paper is organized as follows.  Section 2 briefly explains the improvement of GWO algorithm.  Section 3 illustrates the fault diagnosis method based on *α*-GWO-SVM. Simulation results and performance analysis are provided in Section 4. Contributions of the proposed method are given in Section 5.

## 2. An Improved Grey Wolf Algorithm

Grey Wolf Optimization (GWO) algorithm achieves the optimal outcome in the search of target by simulating the leadership hierarchy and the group hunting mechanism of the grey wolves. It shows advantages such as fast speed of search and satisfactory optimization effect [23]. However, there is still room for improvement in terms of the search strategy for the GWO [24, 25]. Therefore, an improvement is made to the proposed *α* Grey Wolf Optimization (*α*-GWO) algorithm as follows. The wolf pack is still divided into four levels, while default *α*, *β*, and *δ* wolves have strong search capability. Social rank is the highest in the population, and the remaining wolves are denoted as *ω*. The mathematical model for finding prey is expressed as follows:

(1) $$\begin{array}{c} \left\{\begin{array}{c} \overset{⟶}{D}=\left|\overset{⟶}{C}\cdot {\overset{⟶}{X}}_{P}\left(t\right)-\overset{⟶}{X}\left(t\right)\right|,\\ \overset{⟶}{X}\left(t+1\right)={\overset{⟶}{X}}_{P}\left(t\right)-\overset{⟶}{A}\cdot \overset{⟶}{D},\\ \overset{⟶}{A}=2\overset{⟶}{a}\cdot {\overset{⟶}{r}}_{1}-\overset{⟶}{a},\\ \overset{⟶}{C}=2{\overset{⟶}{r}}_{2}, \end{array}\right) \end{array}$$

where *t*represents the number of current iterations, $\overset{⟶}{A}$ and $\overset{⟶}{C}$ denote the synergy coefficients, ${\overset{⟶}{X}}_{P}$ indicates the location of the prey, and $\overset{⟶}{X}$ refers to the current grey wolf position, which linearly decreases from 2 to 0, while ${\overset{⟶}{r}}_{1}$and ${\overset{⟶}{r}}_{2}$ stand for the random vector in [0,1]. In *α*-GWO, a competitive relationship between the head wolves is introduced to improve the global search capability. Corresponding to the search target of the head wolves in each iteration, the fault classification error is taken as the score to obtain alpha score, beta score, and delta score. The head wolf level is rearranged according to the fault error score, and the wolf pack position is updated according to equations (2)–(4):

(2) $$\begin{array}{c} \left\{\begin{array}{c} {\overset{⟶}{D}}_{\alpha }=\left|{\overset{⟶}{C}}_{1}\cdot {\overset{⟶}{X}}_{\alpha }-\overset{⟶}{X}\right|,\\ {\overset{⟶}{X}}_{1}={\overset{⟶}{X}}_{\alpha }-{\overset{⟶}{A}}_{1}\cdot {\overset{⟶}{D}}_{\alpha }, \end{array}\right) \end{array}$$

(3) $$\begin{array}{c} \left\{\begin{array}{c} {\overset{⟶}{D}}_{\beta }=\left|{\overset{⟶}{C}}_{2}\cdot {\overset{⟶}{X}}_{\beta }-\overset{⟶}{X}\right|,\\ {\overset{⟶}{X}}_{2}={\overset{⟶}{X}}_{\beta }-{\overset{⟶}{A}}_{2}\cdot {\overset{⟶}{D}}_{\beta }, \end{array}\right) \end{array}$$

(4) $$\begin{array}{c} \left\{\begin{array}{c} {\overset{⟶}{D}}_{\delta }=\left|{\overset{⟶}{C}}_{3}\cdot {\overset{⟶}{X}}_{\delta }-\overset{⟶}{X}\right|,\\ {\overset{⟶}{X}}_{3}={\overset{⟶}{X}}_{\delta }-{\overset{⟶}{A}}_{3}\cdot {\overset{⟶}{D}}_{\delta }, \end{array}\right) \end{array}$$

where *X* represents the location of the wolf pack, while ${\overset{⟶}{D}}_{\alpha }$, ${\overset{⟶}{D}}_{\beta }$, and ${\overset{⟶}{D}}_{\delta }$ refer to the distance between the current candidate wolf and the best three wolves. When |*A*| > 1, the wolves are dispersed in search of prey; when |*A*| < 1, the wolves start to concentrate on attacking their prey. While ensuring that the selected wolf has the strongest ability in the population, it is adjusted together according to the change of error and the number of current iterations for gradually enhancing the dominant position of the *α* wolf. The improvement is expressed as follows:

(5) $$\begin{array}{c} \left\{\begin{array}{c} {\overset{⟶}{X}}_{1}^{\alpha }={\overset{⟶}{X}}_{1}+\left(\frac{E_{rr}^{\max}-E_{rr}^{t}}{T}\right)\cdot t,\\ \overset{⟶}{X}{\left(t+1\right)}^{\alpha -GWO}=\frac{{\overset{⟶}{X}}_{1}^{\alpha }+{\overset{⟶}{X}}_{2}+{\overset{⟶}{X}}_{3}}{3}, \end{array}\right) \end{array}$$

where *t* represents the number of current iterations, *E*_*rr*_^max^ indicates the maximum classification error, *E*_*rr*_^t^denotes the current classification error, and *T* refers to the total number of times of iteration.

## 3. Fault Diagnosis Method Based on *α*-GWO-SVM

### 3.1. Data Preprocessing

**Figure 1:**
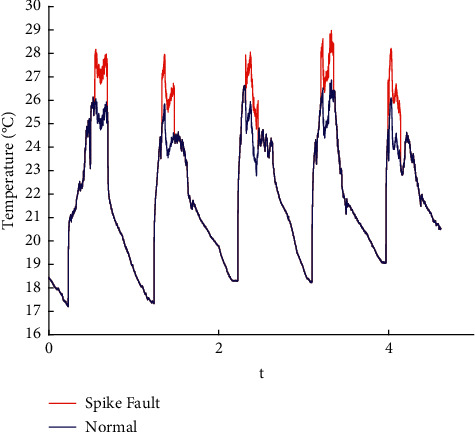
Spike fault.

**Figure 2:**
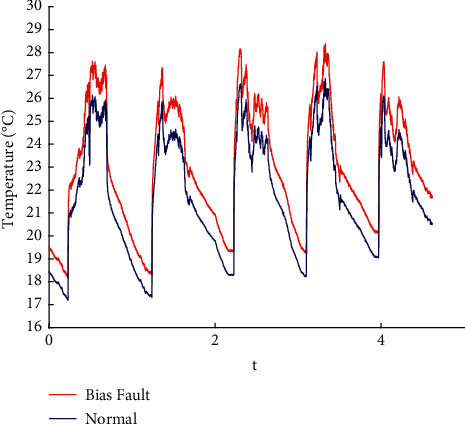
Bias fault.

**Figure 3:**
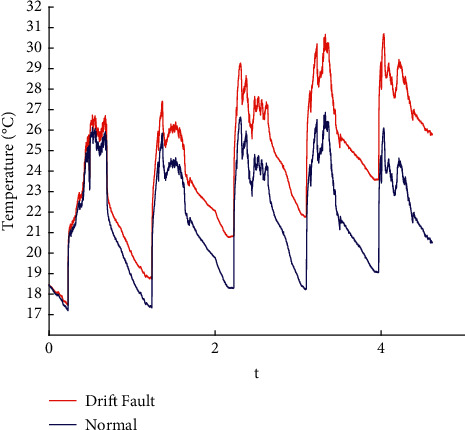
Drift fault.

**Figure 4:**
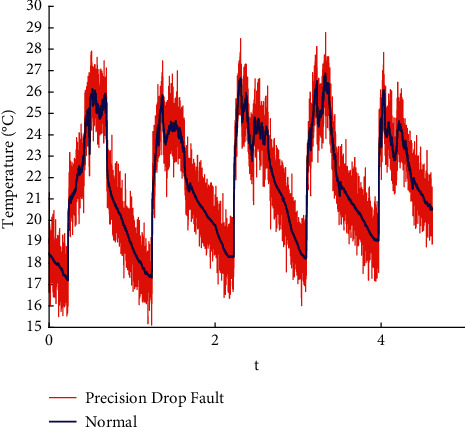
Precision drop fault.

**Figure 5:**
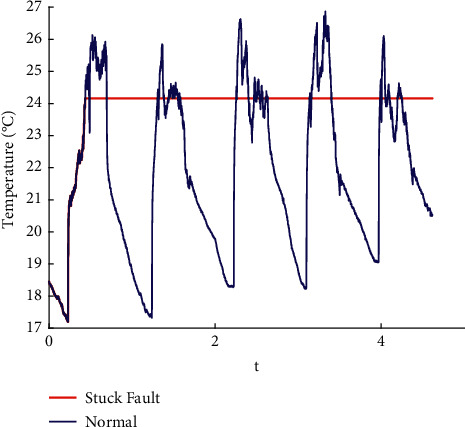
Stuck fault.

**Figure 6:**
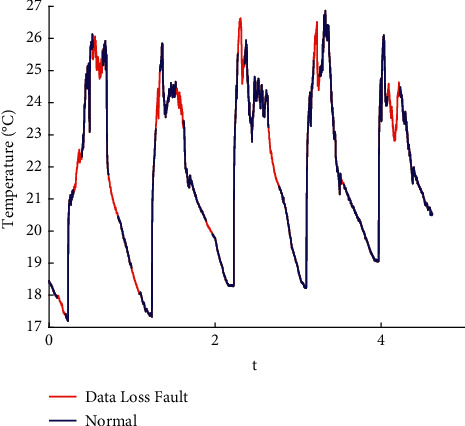
Data loss fault.

**Figure 7:**
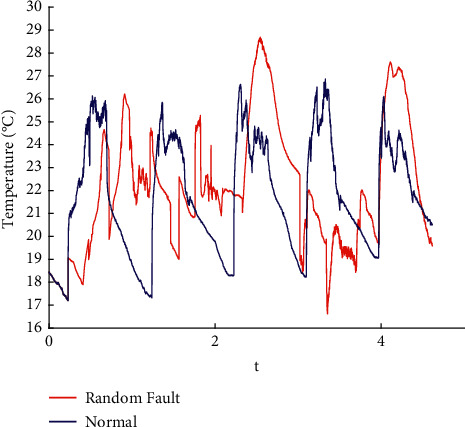
Random fault.

In this paper, the data published online by Intel Labs [26] are used to perform fault injection in line with the existing methods [27]. Spike, bias, drift, precision drop, stuck, data loss, and random fault are injected into the original data. The raw data are shown in the appendix, and the fault sample obtained is shown in Figures 1–7.

### 3.2. Data Feature Extraction

The Kernel Principal Component Analysis (KPCA) is usually conducted to extract features and reduce the dimensionality of nonlinear data [28]. The main steps of KPCA are detailed as follows. Hypothesis {*y*_*i*_} is a collection of time-domain parameters, *i*=1,2,…, *n*, *y*_*i*_is the vector of *m* × 1, and each vector comprises the time-domain parameters. The kernel matrix is calculated according to the following equation:

(6) $$\begin{array}{c} K_{\mu \varphi }=\left(\Phi \left(y_{\mu }\right)\cdot \Phi \left(y_{\varphi }\right)\right). \end{array}$$

According to equation 7 [28], the new kernel matrix KL is obtained by modifying

(7) $$\begin{array}{c} K_{\mu \varphi }⟶K_{\mu \varphi }-\frac{1}{M}\left(\sum_{\omega =1}^{M}K_{\mu \omega }+\sum_{\omega =1}^{M}K_{\omega \varphi }\right)+\frac{1}{M^{2}}\sum_{\omega ,\tau }^{M}K_{\omega \tau }. \end{array}$$

The Jacobian matrix is applied to calculate the eigenvalues of kernel matrix *λ*_1_, *λ*_2_,…, *λ*_*n*_ and eigenvectors *v*_1_, *v*_2_,…, *v*_*n*_, and then the eigenvalues in descending order are sorted. The Gram–Schmidt orthogonalization process is followed to perform unit orthogonalization on the eigenvectors, so as to obtain *v*_1_, *v*_2_,…, *v*_*n*_. Then, components are extracted to obtain the transformation matrix:

(8) $$\begin{array}{c} {\tilde{v}}^{T}={\left[\begin{array}{cccc} {\tilde{v}}_{1}^{T} & {\tilde{v}}_{2}^{T} & \ldots & {\tilde{v}}_{n}^{T} \end{array}\right]}^{T}, \end{array}$$

(9) $$\begin{array}{c} {\tilde{v}}^{T}z^{\prime }=KLz^{\prime }=\tilde{x}. \end{array}$$

Formula 9 is applied to convert the vector through the transformation matrix to $\tilde{x}$, where $\tilde{x}={\left\{{\tilde{x}}_{1},{\tilde{x}}_{2},\ldots ,{\tilde{x}}_{n}\right\}}^{T}$ refers to the extracted principal component vector.

The extracted principal components are fused with the time-domain parameters. The fused features not only contain the overall characteristics of the fault data but also reflect the local characteristics of the fault. Through multiple experimental comparisons, the mean, variance, crest factor, and skewness coefficient are taken as the reference indicators for the local features of the fault data, while the final fusion features are treated as samples.

In total, 342 groups of samples are selected for this experiment, with 242 groups taken as the training dataset and the other 100 groups treated as the testing dataset. Labels 1–8 represent spike, drift, bias, random, stuck, precision drop, data loss fault, and normal, respectively. The training set sample and testing set sample are listed in Table 1.

### 3.3. Establishment of *α*-GWO-SVM Diagnosis Model

SVM provides an effective solution to the limited sample size and nonlinearity [29,30]. During model training and testing, the datasets usually consist of feature vectors and labels. The support vector is obtained by using the feature vector and label in the samples, and then the hyperplanes are established to separate different types of samples. More problems about Support Vector Machine mathematical modeling are detailed in [31]. The “one-to-one,” “one-to-many,” and “many-to-many” methods are used to address multiclassification issues [32].

The labeled fault data samples are used for SVM training, through using the samples and labels to build support vector, and then the hyperplane is established, so as to achieve the division of different types of sample data. In essence, the mathematical model of the multiclass SVM is a convex quadratic programming problem. A critical step is to determine the appropriate kernel function coefficient*γ* and penalty factor *C*. The mathematical modeling process of the multiclass SVM is detailed as follows.

The objective function is constructed for convex quadratic programming

(10) $$\begin{array}{c} \left\{\begin{array}{c} \min Q\left(\alpha \right)=\frac{1}{2}\sum_{i,j=1}^{N}\alpha_{i}\alpha_{j}y_{i}y_{i}K\left(X_{i},X_{j}\right)-\sum_{i=1}^{N}\alpha_{i},\\ \text{S}.\text{t}.\sum_{i,j=1}^{N}\alpha_{i}y_{i}\;=\;0,\\ 0\leq \alpha_{i}\leq C,\;i=1,2,\ldots ,N, \end{array}\right) \end{array}$$

where *α*_*i*_ represents the Lagrange multiplier, *X*_*i*_ and*X*_*j*_ indicate the input vector, *y*_*i*_denotes the category label, and *K*(*X*_*i*_, *X*_*j*_) refers to the kernel function. In fact, not all of the data can be linearly separated to the full, so that the hint loss is taken into consideration:

(11) $$\begin{array}{c} \left\{\begin{array}{c} \underset{\omega ,b,\xi_{i}}{\min}\frac{1}{2}{\left\|\omega \right\|}^{2}+C\sum_{i=1}^{m}\xi_{i},\\ \text{s}.\text{t}.\;y_{i}\left(\omega^{T}x_{i}+b\right)\geq 1-\xi_{i},\\ \xi_{i}\geq 0,\;i=1,2,\ldots ,N, \end{array}\right) \end{array}$$

where *ω* represents the normal plane vector, *ξ*_*i*_ indicates slack variable, with each sample corresponding to one *ξ*_*i*_, representing the degree to which the sample does not meet the constraints, and *C* denotes the penalty factor. The corresponding classification function is expressed as

(12) $$\begin{array}{c} f\left(x\right)=\mathrm{sgn}\left\{\sum_{i=1}^{N}\alpha_{i}^{*}y_{i}K\left(x,x_{i}\right)+b^{*}\right\}, \end{array}$$

where *b*^*∗*^ represents the offset constant. The introduction of kernel function is effective in improving the ability of Support Vector Machine to deal with nonlinearity. In this paper, Gaussian kernel function with superior performance is applied:

(13) $$\begin{array}{c} K\left(x,z\right)=\exp\left(\gamma \left\|x-z\right\|\right). \end{array}$$

It can be seen from equations 11 and 13 that both the penalty factor *C* and kernel function parameter *γ* play an important role in determining the classification performance of Support Vector Machine. The penalty factor *C* determines the degree of fit, and the kernel function parameter *γ* determines the scope of support vector, thus determining the generalization ability of the SVM. Therefore, choosing appropriate parameters is crucial for improving the accuracy of classification.

### 3.4. *α*-GWO Algorithm Optimizes SVM

**Figure 8:**
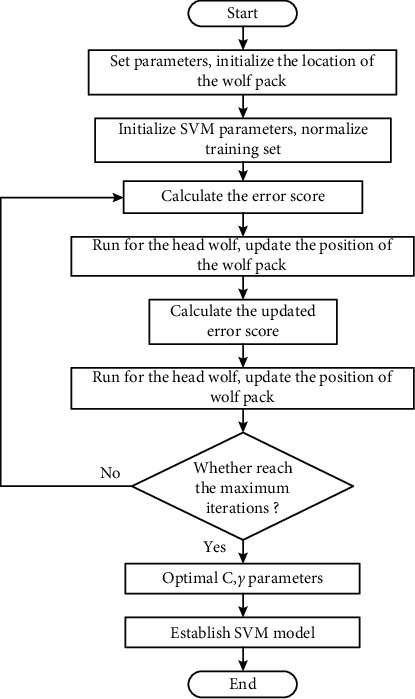
Optimized flowchart.

When the *α*-GWO algorithm is applied to optimize the parameters of SVM, kernel function and penalty factor are the parameters to be optimized. Optimized flow chart is shown in Figure 8. The optimization process is detailed as follows:

- Step 1: set the size of the wolf pack *N*=10, the maximum number of iterations *T*=30, and the search dimension *D*=2, before initializing the location of the wolf pack.
- Step 2: initialize Support Vector Machine(*C*, *γ*) parameters and search range: *C* ∈ [0,100], *γ* ∈ [0.01, 0.15].
- Step 3: calculate the error scores of the three wolves under the current (*C*, *γ*) parameter to rearrange the level of the wolves.
- Step 4: with the smallest classification error of the alpha wolf after the election as the fitness value, update the wolf pack position according to equations (2)–(5).
- Step 5: perform comparison with the fitness value of the previous iteration. If it falls below than the original fitness value, it will not be updated; otherwise, the fitness value will be updated.
- Step 6: perform cyclical calculation until the maximum number of cycles is reached, output (*C*, *γ*) at this time as the optimal parameters of the Support Vector Machine, and construct the SVM model.

**Figure 9:**
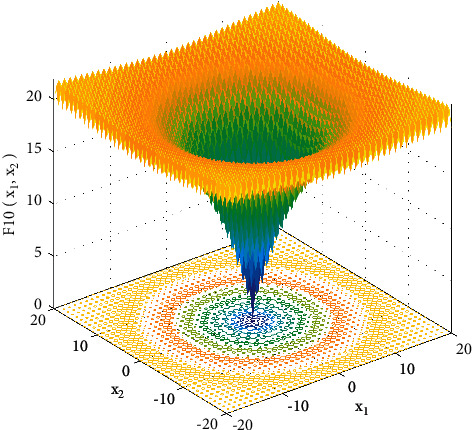
Test function.

In order to verify the effectiveness of the improved algorithm, the function *y*=*x* is selected for testing as shown in Figure 9. Among them, lb=−32, up=32, D=30, and  x=−20:0.5:20.

**Figure 10:**
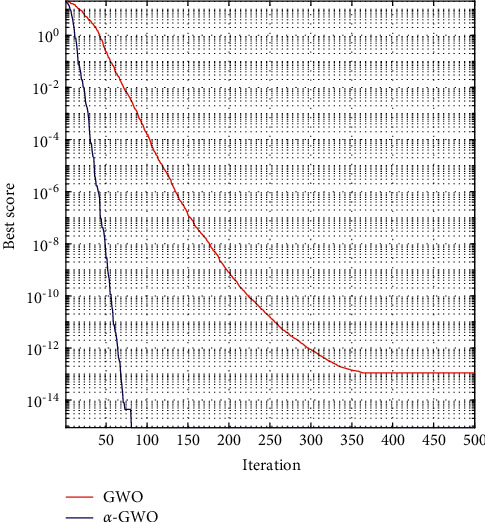
Function optimization curve of GWO and *α*-GWO.

Figure 10 shows the convergence curve after taking logarithm; *α*-GWO has tended to converge after 100 iterations, while GWO has tended to converge after nearly 350 iterations, indicating that the convergence of *α*-GWO is faster than that of GWO. In addition, *α*-GWO is more accurate than GWO in searching for optimal values.

**Figure 11:**
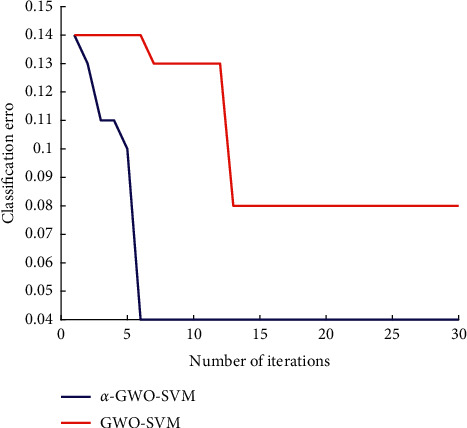
Error curve of GWO-SVM and *α*-GWO-SVM.

The testing dataset comprised of fusion features is inputted into the classifier for testing.  Figure 11 shows the iteration number and error curve of GWO-SVM and *α*-GWO-SVM. After 13 iterations of GWO algorithm, the classification error of SVM reaches 0.08, while *α*-GWO algorithm reveals that the superiority is evident to the original grey wolf algorithm, and the classification error of SVM can reach 0.04 after 6 iterations. Moreover, it can be seen from the classification error that the *α*-GWO algorithm performs better in parameter optimization for the SVM in each iteration, indicating that the improved algorithm has a better capability of optimization.

## 4. Simulation Results and Performance Analysis

### 4.1. Diagnosis Results

#### 4.1.1. Diagnosis Result Comparison of the Before Feature Selection (BFS)

Fault data are obtained by fault injection of the original temperature data as mentioned in the previous section. Then, the mean value, variance, root mean square, peak value, peak value factor, skewness coefficient, and kurtosis coefficient are determined from the fault data, and the principal components of the seven time-domain parameters are extracted. Through the simulation experiment, the peak value, variance, peak factor, skewness coefficient, and the extracted principal component are finally selected and integrated to obtain the final dataset for SVM training and testing.

In this section, two parts of experiments are arranged. The first part is the comparison of the effect of the principal component extraction dataset and the fusion dataset, and the second part is the comparison of the effect of the SVM optimized by different algorithms.

**Figure 12:**
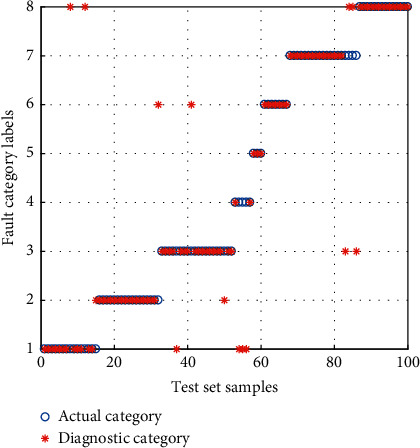
Diagnosis results for the BFS of APSO-SVM.

**Figure 13:**
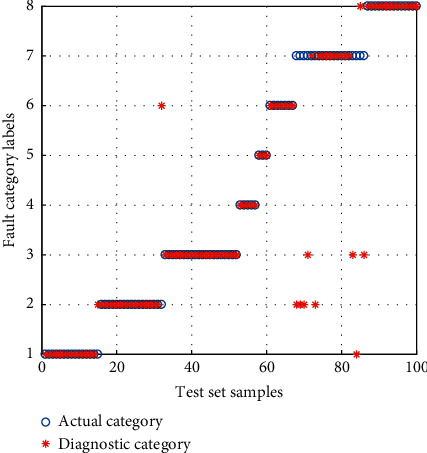
Diagnosis results for the BFS of GWO-SVM.

**Figure 14:**
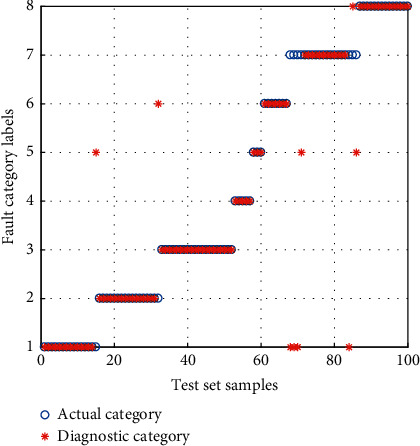
Diagnosis results for the BFS of *α*-GWO-SVM.

The samples of the BFS are inputted into the *α*-GWO-SVM diagnostic model for training and testing. Then, comparison is performed with the GWO-SVM and Adaptive Particle Swarm Optimization SVM (APSO-SVM) diagnostic model. The result of diagnosis is shown in Figures 12–14.

It can be seen from the comparison of diagnostic results that the APSO-SVM and GWO-SVM have misclassified multiple types of faults, suggesting the lowest ability to identify the data loss fault. *α*-GWO-SVM makes a total of 9 sets of errors, and the performance is better than the others. In spite of this, there remain a variety of faults misclassified. It is evidenced that only the use of feature training model extracted by the KPCA leads to the failure of achieving an accurate diagnosis.

#### 4.1.2. Diagnosis Result Comparison of the After Feature Selection (AFS)

**Figure 15:**
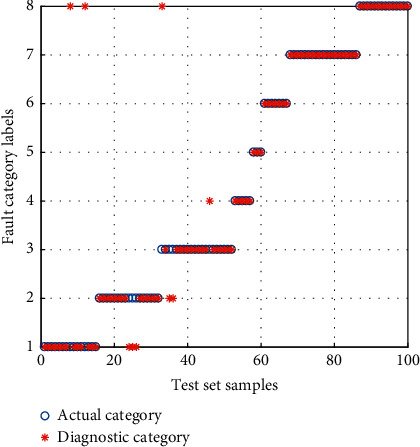
Diagnosis results for the AFS of APSO-SVM.

**Figure 16:**
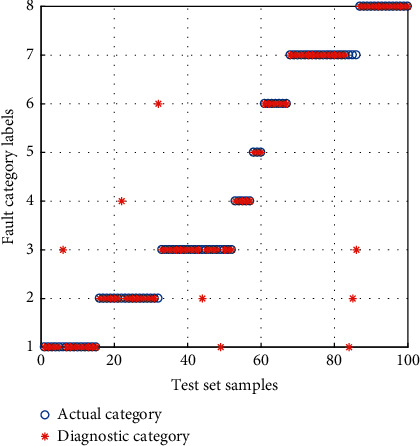
Diagnosis results for the AFS of GWO-SVM.

**Figure 17:**
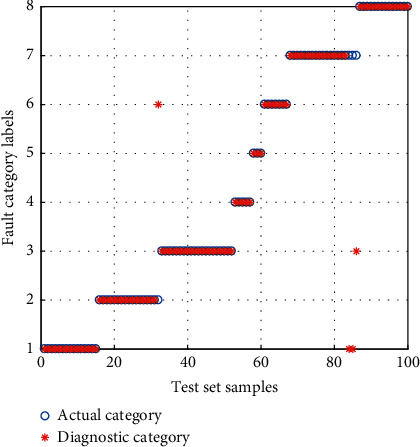
Diagnosis results for the AFS of *α*-GWO-SVM.

The diagnosis results of the AFS are shown in Figures 15–17. According to the analysis of the diagnostic results, the APSO-SVM and GWO-SVM are more accurate, the number of groups that misclassify samples is smaller, and the classification performance has been significantly improved. It is demonstrated that the fused features can be effective in improving the reliability of diagnosis.

### 4.2. Comparative Analysis of Classifier Performance

Since this experiment is a multiclassification problem with the unbalanced distribution of samples [33], precision and kappa coefficient are taken into consideration for evaluating the performance of the classifier. Among them, precision represents the capability of classifier to distinguish each type of sample correctly, and a greater value indicates a better performance of the classification possess. The kappa coefficient evidences the consistency of diagnostic results produced by the classifier with the actual category of samples [34]. Besides, a greater value indicates a better performance of the classification possesses. The mathematical equations of precision and kappa coefficient are expressed as follows:

*Precision*. Calculate the precision of each label separately, with the unweighted average taken.

(14) $$\begin{array}{c} P=\frac{T_{P}}{T_{P}+F_{P}}, \end{array}$$

where *T*_*P*_ represents the number of true positive and *F*_*P*_ refers to the number of false positive. *T*_*P*_ indicates the capability of the classifier to diagnose a sample accurately, according to their respective class. *F*_*P*_ means that the classifier diagnoses a sample inaccurately.

(15) $$\begin{array}{c} \left\{\begin{array}{c} K=\frac{P_{0}-P_{e}}{1-P_{e}},\\ P_{e}=\frac{\sum \left(a_{1}\cdot b_{1}+a_{2}\cdot b_{2}+\cdots +a_{c}\cdot b_{c}\right)}{n^{2}}, \end{array}\right) \end{array}$$

where *P*_0_ is the classification accuracy for all the samples, *a*_*c*_ is the number of real samples of class *c*, *b*_*c*_ is the number of diagnosed samples of class *c*, and *n* is the total number of samples.

**Figure 18:**
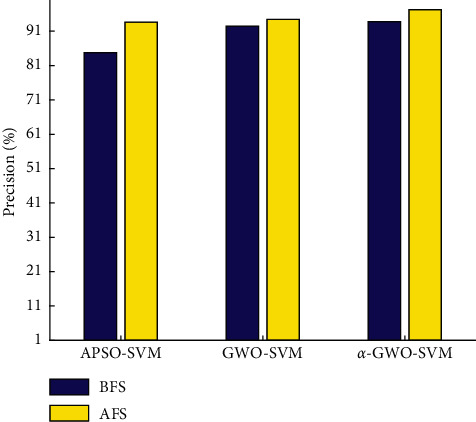
Comparison of precision.

**Figure 19:**
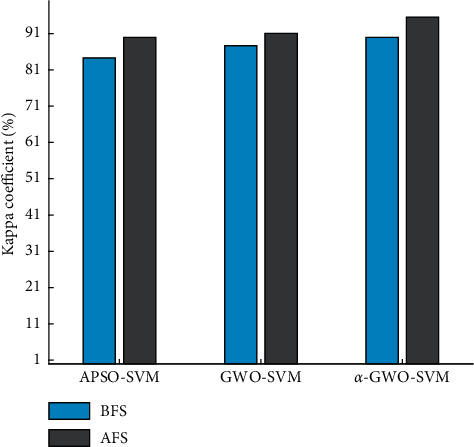
Comparison of kappa coefficient.

The performance index comparison results of the classifier are shown in Figures 18 and 19 and Tables 2 and 3, respectively. As for the BFS, the precision of *α*-GWO-SVM reaches 93.83%, while the kappa coefficient reaches 89.91%. Besides, there are only 9 groups of samples which are wrong, indicating the best classification performance. In contrast to the GWO algorithm, precision has improved by 1.32%, while the kappa coefficient has increased by 2.24%, suggesting that the improved algorithm performs better in optimizing the parameters of Support Vector Machine.

With regard to the AFS, the classifier produces an excellent performance. Precision of *α*-GWO-SVM is 97.29% and the kappa coefficient is 95.52%. Besides, there are as few as 4 sets of samples getting misclassified. As compared to the BFS, the precision is improved by 2.82% and kappa coefficient is increased by 4.49%, suggesting that the feature fusion is effective in enhancing the reliability of diagnosis.

## 5. Conclusion

The considerable contributions of the presented sensor fault diagnosis method in comparison to the previous approaches are summarized as follows:

- In order to improve the accuracy of sensor fault diagnosis, an integrated sensor fault diagnosis approach based on the combination of data-driven and intelligent diagnosis is proposed in this paper. According to the results, this method is capable to achieve an accurate diagnosis of sensor fault when the failure intensity stays low.
- In order to fully extract the valuable information from the fault data, a method of feature extraction is put forward based on the fusion of KPCA and time-domain parameters, and experiments are conducted to demonstrate that the fusion feature improves the accuracy of diagnosis effectively.
- In addition, *α*-GWO algorithm is proposed to optimize the parameters of SVM, thus enhancing the generalization ability of SVM. Through multiple comparison experiments and the analysis of performance indicators such as the precision and kappa coefficient, it is concluded that as compared to the other intelligent diagnosis algorithms based on SVM, the *α*-GWO-SVM diagnostic method produces a better classification performance, and that the proposed method is effective in improving the reliability of diagnosis. In the future, the focus of research will be on the universality of this method proposed.

## Figures and Tables

**Table 1:** Distribution of sample data.

Fault type	Training set	Testing set
Spike fault	60 groups	15 groups
Drift fault	15 groups	17 groups
Bias fault	28 groups	20 groups
Random fault	17 groups	5 groups
Stuck fault	57 groups	3 groups
Erratic fault	23 groups	7 groups
Data loss fault	25 groups	19 groups

**Table 2:** Comparison of precision and kappa coefficient.

Diagnosis model	APSO-SVM	GWO-SVM	*α*-GWO-SVM
Precision of the BFS	84.76%	92.51%	93.83%
Precision of the AFS	93.63%	94.47%	97.29%
Kappa coefficient of the BFS	84.30%	87.67%	89.91%
Kappa coefficient of the AFS	89.91%	91.03%	95.52%

**Table 3:** Comparison of diagnosis results after feature fusion.

Diagnosis model	APSO-SVM	GWO-SVM	*α*-GWO-SVM
Number of misclassification groups of the BFS	14 groups	11 groups	9 groups
Number of misclassification groups of the AFS	9 groups	8 groups	4 groups

## Data Availability

The data used to support the findings of this study are included within the supplementary information file.

## Supplementary Materials

Supplementary Materials “Experimental data.docx” contains the dataset used for the experiment in this paper.
