# Supplementary material for: Sensor Fault Diagnosis Method Based on α-Grey Wolf Optimization-Support Vector Machine
Source: Comput Intell Neurosci. 2021 Sep 10;2021:1956394. doi: 10.1155/2021/1956394 (PMC8448603; doi:10.1155/2021/1956394)
Supplement: Supplementary Materials — “Experimental data.docx” contains the dataset used for the experiment in this paper. [file 1956394.f1.docx]

| 8.49E-05 | -0.000665319 | 0.0011822 | -0.000125097 | 19.79663375 | 6.107340976 | 1.29017677 | 2.911161685 | 1 |
| --- | --- | --- | --- | --- | --- | --- | --- | --- |
| 6.14E-05 | -0.000665844 | 0.001176085 | -0.000124787 | 19.79440989 | 6.088261861 | 1.289347802 | 2.909736338 | 1 |
| -0.0002286 | -0.000225872 | 0.001531635 | -9.81E-05 | 19.8062595 | 6.18475755 | 1.288777835 | 2.905540578 | 1 |
| 0.000134282 | -0.000676495 | 0.001202507 | -0.000110215 | 19.80737143 | 6.194276218 | 1.289192143 | 2.906020947 | 1 |
| 0.000141651 | -0.000676302 | 0.001204433 | -0.000110298 | 19.80807195 | 6.200283127 | 1.289453113 | 2.906334419 | 1 |
| 0.000148674 | -0.000676122 | 0.001206269 | -0.000110377 | 19.80873911 | 6.206011287 | 1.289701624 | 2.906640728 | 1 |
| 0.000163792 | -0.000675742 | 0.001210224 | -0.000110545 | 19.8101735 | 6.218350925 | 1.290235818 | 2.907324858 | 1 |
| 0.000181883 | -0.000675306 | 0.001214962 | -0.000110745 | 19.81188699 | 6.233134648 | 1.290873764 | 2.908187629 | 1 |
| -0.00320544 | 0.000970225 | -0.00019159 | -0.000932386 | 19.81937294 | 0.295587014 | 1.291168323 | 2.906968077 | 1 |
| 5.86E-05 | -0.000672096 | 0.001179044 | -0.000117032 | 19.79717124 | 6.109278269 | 1.287883599 | 2.90581683 | 1 |
| 0.000401834 | -0.000689516 | 0.00128438 | -8.94E-05 | 19.84157464 | 6.485350269 | 1.294439405 | 2.907867337 | 1 |
| 0.000342887 | -0.000690931 | 0.001268762 | -8.90E-05 | 19.83601498 | 6.436576942 | 1.292374985 | 2.905757924 | 1 |
| 0.000214497 | -0.000694709 | 0.001234925 | -8.79E-05 | 19.82378373 | 6.331015018 | 1.287825725 | 2.90295909 | 1 |
| -0.00271726 | 0.000509625 | -0.000479836 | -0.000930089 | 19.83823885 | 0.456026975 | 1.29320101 | 2.906539971 | 1 |
| 0.000390015 | -0.000689784 | 0.001281244 | -8.93E-05 | 19.84046271 | 6.475556072 | 1.294026692 | 2.90740442 | 1 |
| 0.000191346 | -0.000695495 | 0.001228851 | -8.77E-05 | 19.82155987 | 6.312078897 | 1.286997473 | 2.9027287 | 1 |
| 0.000181294 | -0.000695847 | 0.001226216 | -8.76E-05 | 19.82059249 | 6.303866361 | 1.286637077 | 2.902655693 | 1 |
| -0.00289432 | 0.000504381 | -0.000526519 | -0.000928602 | 19.82137084 | 0.310472972 | 1.286927056 | 2.902713134 | 1 |
| -0.00015373 | -0.000244349 | 0.001562638 | -7.58E-05 | 19.82214919 | 6.317089269 | 1.287216993 | 2.902781277 | 1 |
| 8.49E-05 | -0.000665319 | 0.0011822 | -0.000125097 | 19.79663375 | 6.107340976 | 1.29017677 | 2.911161685 | 1 |
| 6.14E-05 | -0.000665844 | 0.001176085 | -0.000124787 | 19.79440989 | 6.088261861 | 1.289347802 | 2.909736338 | 1 |
| -0.0002286 | -0.000225872 | 0.001531635 | -9.81E-05 | 19.8062595 | 6.18475755 | 1.288777835 | 2.905540578 | 1 |
| 0.000134282 | -0.000676495 | 0.001202507 | -0.000110215 | 19.80737143 | 6.194276218 | 1.289192143 | 2.906020947 | 1 |
| 0.000141651 | -0.000676302 | 0.001204433 | -0.000110298 | 19.80807195 | 6.200283127 | 1.289453113 | 2.906334419 | 1 |
| -0.00320544 | 0.000970225 | -0.00019159 | -0.000932386 | 19.81937294 | 0.295587014 | 1.291168323 | 2.906968077 | 1 |
| 5.86E-05 | -0.000672096 | 0.001179044 | -0.000117032 | 19.79717124 | 6.109278269 | 1.287883599 | 2.90581683 | 1 |
| 0.000401834 | -0.000689516 | 0.00128438 | -8.94E-05 | 19.84157464 | 6.485350269 | 1.294439405 | 2.907867337 | 1 |
| 0.000342887 | -0.000690931 | 0.001268762 | -8.90E-05 | 19.83601498 | 6.436576942 | 1.292374985 | 2.905757924 | 1 |
| 0.000214497 | -0.000694709 | 0.001234925 | -8.79E-05 | 19.82378373 | 6.331015018 | 1.287825725 | 2.90295909 | 1 |
| 0.002413302 | -0.003565101 | -0.000919083 | -0.000186452 | 19.8101735 | 6.218350925 | 1.290235818 | 2.907324858 | 1 |
| 0.002436552 | -0.003571299 | -0.000919238 | -0.000186823 | 19.81188699 | 6.233134648 | 1.290873764 | 2.908187629 | 1 |
| 0.002450152 | -0.003574935 | -0.000919328 | -0.00018704 | 19.81288772 | 6.24179059 | 1.291246254 | 2.908714325 | 1 |
| 0.001768089 | -0.000901084 | -0.001849139 | 0.000186306 | 19.57511731 | 4.433886858 | 1.249204802 | 2.8676034 | 1 |
| 0.000148674 | -0.000676122 | 0.001206269 | -0.000110377 | 19.80873911 | 6.206011287 | 1.289701624 | 2.906640728 | 1 |
| 0.000163792 | -0.000675742 | 0.001210224 | -0.000110545 | 19.8101735 | 6.218350925 | 1.290235818 | 2.907324858 | 1 |
| 0.000181883 | -0.000675306 | 0.001214962 | -0.000110745 | 19.81188699 | 6.233134648 | 1.290873764 | 2.908187629 | 1 |
| 0.000192465 | -0.000675059 | 0.001217735 | -0.000110861 | 19.81288772 | 6.24179059 | 1.291246254 | 2.908714325 | 1 |
| 0.000134282 | -0.000676495 | 0.001202507 | -0.000110215 | 19.80737143 | 6.194276218 | 1.289192143 | 2.906020947 | 1 |
| 0.000141651 | -0.000676302 | 0.001204433 | -0.000110298 | 19.80807195 | 6.200283127 | 1.289453113 | 2.906334419 | 1 |
| -0.00268493 | 0.000301902 | -0.000683893 | -0.00095016 | 19.81937294 | 0.295587014 | 1.291168323 | 2.906968077 | 1 |
| 0.002270436 | -0.00351305 | -0.000914566 | -0.000191683 | 19.79717124 | 6.109278269 | 1.287883599 | 2.90581683 | 1 |
| 0.002743359 | -0.003697197 | -0.00093219 | -0.000168382 | 19.84157464 | 6.485350269 | 1.294439405 | 2.907867337 | 1 |
| 0.002667314 | -0.003676617 | -0.000931586 | -0.000167359 | 19.83601498 | 6.436576942 | 1.292374985 | 2.905757924 | 1 |
| 0.002501755 | -0.003632582 | -0.00093016 | -0.000165082 | 19.82378373 | 6.331015018 | 1.287825725 | 2.90295909 | 1 |
| -0.00249259 | 0.000221145 | -0.00069234 | -0.000937744 | 19.83823885 | 0.456026975 | 1.29320101 | 2.906539971 | 1 |
| 0.002728111 | -0.003693052 | -0.000932071 | -0.000168178 | 19.84046271 | 6.475556072 | 1.294026692 | 2.90740442 | 1 |
| 0.002471912 | -0.00362476 | -0.000929886 | -0.000164665 | 19.82155987 | 6.312078897 | 1.286997473 | 2.9027287 | 1 |
| 0.002458954 | -0.003621375 | -0.000929766 | -0.000164484 | 19.82059249 | 6.303866361 | 1.286637077 | 2.902655693 | 1 |
| -0.00272098 | 0.000281822 | -0.000690463 | -0.00093451 | 19.82137084 | 0.310472972 | 1.286927056 | 2.902713134 | 1 |
| 0.002479812 | -0.003626827 | -0.000929959 | -0.000164776 | 19.82214919 | 6.317089269 | 1.287216993 | 2.902781277 | 1 |
| 0.002296421 | -0.003505867 | -0.000911111 | -0.000199735 | 19.79663375 | 6.107340976 | 1.29017677 | 2.911161685 | 1 |
| 0.002266301 | -0.003497871 | -0.000910943 | -0.000199205 | 19.79440989 | 6.088261861 | 1.289347802 | 2.909736338 | 1 |
| 0.002360371 | -0.003551068 | -0.000918717 | -0.000185601 | 19.8062595 | 6.18475755 | 1.288777835 | 2.905540578 | 1 |
| 0.002375383 | -0.003555037 | -0.000918822 | -0.000185843 | 19.80737143 | 6.194276218 | 1.289192143 | 2.906020947 | 1 |
| 0.002384851 | -0.003557544 | -0.000918888 | -0.000185995 | 19.80807195 | 6.200283127 | 1.289453113 | 2.906334419 | 1 |
| -0.00268493 | 0.000301902 | -0.000683893 | -0.00095016 | 19.81937294 | 0.295587014 | 1.291168323 | 2.906968077 | 1 |
| 0.002270436 | -0.00351305 | -0.000914566 | -0.000191683 | 19.79717124 | 6.109278269 | 1.287883599 | 2.90581683 | 1 |
| 0.002743359 | -0.003697197 | -0.00093219 | -0.000168382 | 19.84157464 | 6.485350269 | 1.294439405 | 2.907867337 | 1 |
| 0.002667314 | -0.003676617 | -0.000931586 | -0.000167359 | 19.83601498 | 6.436576942 | 1.292374985 | 2.905757924 | 1 |
| 8.49E-05 | -0.000665319 | 0.0011822 | -0.000125097 | 19.79663375 | 6.107340976 | 1.29017677 | 2.911161685 | 1 |
| 6.14E-05 | -0.000665844 | 0.001176085 | -0.000124787 | 19.79440989 | 6.088261861 | 1.289347802 | 2.909736338 | 1 |
| -0.0002286 | -0.000225872 | 0.001531635 | -9.81E-05 | 19.8062595 | 6.18475755 | 1.288777835 | 2.905540578 | 1 |
| 0.000134282 | -0.000676495 | 0.001202507 | -0.000110215 | 19.80737143 | 6.194276218 | 1.289192143 | 2.906020947 | 1 |
| 0.000141651 | -0.000676302 | 0.001204433 | -0.000110298 | 19.80807195 | 6.200283127 | 1.289453113 | 2.906334419 | 1 |
| 0.000148674 | -0.000676122 | 0.001206269 | -0.000110377 | 19.80873911 | 6.206011287 | 1.289701624 | 2.906640728 | 1 |
| 0.000163792 | -0.000675742 | 0.001210224 | -0.000110545 | 19.8101735 | 6.218350925 | 1.290235818 | 2.907324858 | 1 |
| 0.000181294 | -0.000695847 | 0.001226216 | -8.76E-05 | 19.82059249 | 6.303866361 | 1.286637077 | 2.902655693 | 1 |
| -0.00271726 | 0.000509625 | -0.000479836 | -0.000930089 | 19.83823885 | 0.456026975 | 1.29320101 | 2.906539971 | 1 |
| 0.000390015 | -0.000689784 | 0.001281244 | -8.93E-05 | 19.84046271 | 6.475556072 | 1.294026692 | 2.90740442 | 1 |
| 0.000191346 | -0.000695495 | 0.001228851 | -8.77E-05 | 19.82155987 | 6.312078897 | 1.286997473 | 2.9027287 | 1 |
| 0.000181294 | -0.000695847 | 0.001226216 | -8.76E-05 | 19.82059249 | 6.303866361 | 1.286637077 | 2.902655693 | 1 |
| -0.00289432 | 0.000504381 | -0.000526519 | -0.000928602 | 19.82137084 | 0.310472972 | 1.286927056 | 2.902713134 | 1 |
| -0.00015373 | -0.000244349 | 0.001562638 | -7.58E-05 | 19.82214919 | 6.317089269 | 1.287216993 | 2.902781277 | 1 |
| 0.000192465 | -0.000675059 | 0.001217735 | -0.000110861 | 19.81288772 | 6.24179059 | 1.291246254 | 2.908714325 | 1 |
| 0.000129028 | 0.001204235 | -0.000298242 | 0.000241797 | 19.57511731 | 4.433886858 | 1.249204802 | 2.8676034 | 1 |
| 0.000207091 | 0.000955497 | 0.000538237 | 1.28E-05 | 21.39056648 | 5.038235009 | 1.210524725 | 1.941343393 | 2 |
| 0.000318387 | 0.001072148 | 0.000516266 | 1.57E-05 | 21.52596648 | 5.033527165 | 1.207069314 | 1.958249987 | 2 |
| 0.000379845 | 0.001134667 | 0.000507041 | 1.77E-05 | 21.59840548 | 5.036023311 | 1.205231605 | 1.969300842 | 2 |
| 0.000335482 | 0.001089671 | 0.000513504 | 1.62E-05 | 21.54627648 | 5.033874366 | 1.2065533 | 1.961208163 | 2 |
| 0.000307049 | 0.001060468 | 0.000518184 | 1.53E-05 | 21.51242648 | 5.03344836 | 1.207413656 | 1.956338854 | 2 |
| 0.000379845 | 0.001134667 | 0.000507041 | 1.77E-05 | 21.59840548 | 5.036023311 | 1.205231605 | 1.969300842 | 2 |
| 0.000335482 | 0.001089671 | 0.000513504 | 1.62E-05 | 21.54627648 | 5.033874366 | 1.2065533 | 1.961208163 | 2 |
| 0.000307049 | 0.001060468 | 0.000518184 | 1.53E-05 | 21.51242648 | 5.03344836 | 1.207413656 | 1.956338854 | 2 |
| 0.002512234 | -0.001524273 | -0.001482096 | -5.19E-05 | 21.63902548 | 5.038952708 | 1.204204446 | 1.97610155 | 2 |
| 0.002361667 | -0.001630933 | -0.001443411 | -5.56E-05 | 21.48534648 | 5.033657145 | 1.208103139 | 1.952663307 | 2 |
| 0.002324293 | -0.001660892 | -0.001433206 | -5.64E-05 | 21.44472648 | 5.034886302 | 1.209139362 | 1.947517571 | 2 |
| 0.002287934 | -0.001691591 | -0.001423008 | -5.70E-05 | 21.40410648 | 5.037214636 | 1.210177984 | 1.942813393 | 2 |
| 0.00227604 | -0.001701988 | -0.001419611 | -5.72E-05 | 21.39056648 | 5.038235009 | 1.210524725 | 1.941343393 | 2 |
| 0.002400054 | -0.001601712 | -0.001453625 | -5.48E-05 | 21.52596648 | 5.033527165 | 1.207069314 | 1.958249987 | 2 |
| 0.001778032 | -0.002444865 | -0.001224886 | -4.98E-05 | 20.60524648 | 5.306382443 | 1.231092924 | 1.931883499 | 2 |
| 0.002471027 | -0.001551437 | -0.00147186 | -5.30E-05 | 21.59840548 | 5.036023311 | 1.205231605 | 1.969300842 | 2 |
| 0.002419628 | -0.001587379 | -0.001458735 | -5.43E-05 | 21.54627648 | 5.033874366 | 1.2065533 | 1.961208163 | 2 |
| 0.002387145 | -0.00161137 | -0.00145022 | -5.51E-05 | 21.51242648 | 5.03344836 | 1.207413656 | 1.956338854 | 2 |
| 0.002471027 | -0.001551437 | -0.00147186 | -5.30E-05 | 21.59840548 | 5.036023311 | 1.205231605 | 1.969300842 | 2 |
| 0.002419628 | -0.001587379 | -0.001458735 | -5.43E-05 | 21.54627648 | 5.033874366 | 1.2065533 | 1.961208163 | 2 |
| 0.002387145 | -0.00161137 | -0.00145022 | -5.51E-05 | 21.51242648 | 5.03344836 | 1.207413656 | 1.956338854 | 2 |
| 0.000207091 | 0.000955497 | 0.000538237 | 1.28E-05 | 21.39056648 | 5.038235009 | 1.210524725 | 1.941343393 | 2 |
| 0.000318387 | 0.001072148 | 0.000516266 | 1.57E-05 | 21.52596648 | 5.033527165 | 1.207069314 | 1.958249987 | 2 |
| 0.000379845 | 0.001134667 | 0.000507041 | 1.77E-05 | 21.59840548 | 5.036023311 | 1.205231605 | 1.969300842 | 2 |
| 0.000335482 | 0.001089671 | 0.000513504 | 1.62E-05 | 21.54627648 | 5.033874366 | 1.2065533 | 1.961208163 | 2 |
| 0.000307049 | 0.001060468 | 0.000518184 | 1.53E-05 | 21.51242648 | 5.03344836 | 1.207413656 | 1.956338854 | 2 |
| 0.000379845 | 0.001134667 | 0.000507041 | 1.77E-05 | 21.59840548 | 5.036023311 | 1.205231605 | 1.969300842 | 2 |
| 0.000414906 | 0.00116974 | 0.000502625 | 1.91E-05 | 21.63902548 | 5.038952708 | 1.204204446 | 1.97610155 | 2 |
| 0.000284513 | 0.001037118 | 0.000522206 | 1.47E-05 | 21.48534648 | 5.033657145 | 1.208103139 | 1.952663307 | 2 |
| 0.000251057 | 0.001002115 | 0.000528702 | 1.38E-05 | 21.44472648 | 5.034886302 | 1.209139362 | 1.947517571 | 2 |
| 0.000218014 | 0.000967145 | 0.000535759 | 1.30E-05 | 21.40410648 | 5.037214636 | 1.210177984 | 1.942813393 | 2 |
| -0.00035171 | 0.000290492 | 0.000790501 | 2.22E-05 | 20.60524648 | 5.306382443 | 1.231092924 | 1.931883499 | 2 |
| 0.002635461 | 0.000511811 | 0.001372754 | 0.000243537 | 21.51600417 | 8.017711248 | 1.25310329 | 2.155240943 | 3 |
| 0.003259529 | 0.000761364 | 0.001368394 | 0.000262626 | 21.88967296 | 8.298079403 | 1.252537152 | 2.155173042 | 3 |
| 0.001241888 | -4.30E-05 | 0.00138619 | 0.000213116 | 20.6978218 | 7.421067104 | 1.251800858 | 2.154421145 | 3 |
| 0.003232849 | 0.000748655 | 0.00137072 | 0.000260753 | 21.8691107 | 8.28700856 | 1.252994598 | 2.154627502 | 3 |
| 0.003163639 | 0.00072039 | 0.001370932 | 0.000258769 | 21.82792455 | 8.256201223 | 1.252984354 | 2.155846349 | 3 |
| 0.002810243 | 0.000582345 | 0.001371254 | 0.000248444 | 21.62056177 | 8.094681708 | 1.253025116 | 2.154726235 | 3 |
| 0.002651398 | 0.000519206 | 0.001372841 | 0.00024397 | 21.52565049 | 8.024474021 | 1.253143909 | 2.153585009 | 3 |
| 0.002635461 | 0.000511811 | 0.001372754 | 0.000243537 | 21.51600417 | 8.017711248 | 1.25310329 | 2.155240943 | 3 |
| 0.003259529 | 0.000761364 | 0.001368394 | 0.000262626 | 21.88967296 | 8.298079403 | 1.252537152 | 2.155173042 | 3 |
| 0.001241888 | -4.30E-05 | 0.00138619 | 0.000213116 | 20.6978218 | 7.421067104 | 1.251800858 | 2.154421145 | 3 |
| 0.003232849 | 0.000748655 | 0.00137072 | 0.000260753 | 21.8691107 | 8.28700856 | 1.252994598 | 2.154627502 | 3 |
| 0.003163639 | 0.00072039 | 0.001370932 | 0.000258769 | 21.82792455 | 8.256201223 | 1.252984354 | 2.155846349 | 3 |
| 0.002810243 | 0.000582345 | 0.001371254 | 0.000248444 | 21.62056177 | 8.094681708 | 1.253025116 | 2.154726235 | 3 |
| 0.005830955 | -0.003355642 | -0.001518632 | 0.000145609 | 21.60044464 | 8.084373934 | 1.252986332 | 2.153883565 | 3 |
| 0.005770248 | -0.003361582 | -0.001509028 | 0.000141585 | 21.56755097 | 8.054651451 | 1.253582649 | 2.154934946 | 3 |
| 0.005730106 | -0.003374942 | -0.00150463 | 0.000143432 | 21.54671345 | 8.046638735 | 1.253112374 | 2.154559816 | 3 |
| 0.005683636 | -0.003377771 | -0.001498972 | 0.00014185 | 21.52565049 | 8.024474021 | 1.253143909 | 2.153585009 | 3 |
| 0.005664901 | -0.003381555 | -0.0014964 | 0.00014151 | 21.51600417 | 8.017711248 | 1.25310329 | 2.155240943 | 3 |
| 0.00638664 | -0.003258162 | -0.00159369 | 0.00015737 | 21.88967296 | 8.298079403 | 1.252537152 | 2.155173042 | 3 |
| 0.004065807 | -0.003671087 | -0.001287519 | 0.000117882 | 20.6978218 | 7.421067104 | 1.251800858 | 2.154421145 | 3 |
| 0.002651398 | 0.000519206 | 0.001372841 | 0.00024397 | 21.52565049 | 8.024474021 | 1.253143909 | 2.153585009 | 3 |
| 0.002635461 | 0.000511811 | 0.001372754 | 0.000243537 | 21.51600417 | 8.017711248 | 1.25310329 | 2.155240943 | 3 |
| 0.003259529 | 0.000761364 | 0.001368394 | 0.000262626 | 21.88967296 | 8.298079403 | 1.252537152 | 2.155173042 | 3 |
| 0.001241888 | -4.30E-05 | 0.00138619 | 0.000213116 | 20.6978218 | 7.421067104 | 1.251800858 | 2.154421145 | 3 |
| 0.002651398 | 0.000519206 | 0.001372841 | 0.00024397 | 21.52565049 | 8.024474021 | 1.253143909 | 2.153585009 | 3 |
| 0.002635461 | 0.000511811 | 0.001372754 | 0.000243537 | 21.51600417 | 8.017711248 | 1.25310329 | 2.155240943 | 3 |
| 0.003259529 | 0.000761364 | 0.001368394 | 0.000262626 | 21.88967296 | 8.298079403 | 1.252537152 | 2.155173042 | 3 |
| 0.001241888 | -4.30E-05 | 0.00138619 | 0.000213116 | 20.6978218 | 7.421067104 | 1.251800858 | 2.154421145 | 3 |
| 0.007869739 | -0.004406914 | -0.001658614 | 0.000381186 | 21.8691107 | 10.04000316 | 1.252994598 | 2.154627502 | 3 |
| 0.00778951 | -0.004420888 | -0.001647879 | 0.000379562 | 21.82792455 | 10.00918558 | 1.252984354 | 2.155846349 | 3 |
| 0.007380229 | -0.004486426 | -0.001594119 | 0.000371076 | 21.62056177 | 9.847706824 | 1.253025116 | 2.154726235 | 3 |
| 0.007197481 | -0.004518505 | -0.001569597 | 0.000367404 | 21.52565049 | 9.77761793 | 1.253143909 | 2.153585009 | 3 |
| 0.007178722 | -0.004522262 | -0.001567023 | 0.000367058 | 21.51600417 | 9.770814538 | 1.25310329 | 2.155240943 | 3 |
| 0.00638664 | -0.003258162 | -0.00159369 | 0.00015737 | 21.88967296 | 8.298079403 | 1.252537152 | 2.155173042 | 3 |
| 0.004065807 | -0.003671087 | -0.001287519 | 0.000117882 | 20.6978218 | 7.421067104 | 1.251800858 | 2.154421145 | 3 |
| 0.006356415 | -0.003266287 | -0.001587989 | 0.000155615 | 21.8691107 | 8.28700856 | 1.252994598 | 2.154627502 | 3 |
| 0.006276147 | -0.003280267 | -0.001577256 | 0.000153996 | 21.82792455 | 8.256201223 | 1.252984354 | 2.155846349 | 3 |
| 0.00586659 | -0.003345771 | -0.001523497 | 0.000145527 | 21.62056177 | 8.094681708 | 1.253025116 | 2.154726235 | 3 |
| 0.002651398 | 0.000519206 | 0.001372841 | 0.00024397 | 21.52565049 | 8.024474021 | 1.253143909 | 2.153585009 | 3 |
| 0.002635461 | 0.000511811 | 0.001372754 | 0.000243537 | 21.51600417 | 8.017711248 | 1.25310329 | 2.155240943 | 3 |
| 0.003259529 | 0.000761364 | 0.001368394 | 0.000262626 | 21.88967296 | 8.298079403 | 1.252537152 | 2.155173042 | 3 |
| 0.001241888 | -4.30E-05 | 0.00138619 | 0.000213116 | 20.6978218 | 7.421067104 | 1.251800858 | 2.154421145 | 3 |
| 0.003232849 | 0.000748655 | 0.00137072 | 0.000260753 | 21.8691107 | 8.28700856 | 1.252994598 | 2.154627502 | 3 |
| 0.003163639 | 0.00072039 | 0.001370932 | 0.000258769 | 21.82792455 | 8.256201223 | 1.252984354 | 2.155846349 | 3 |
| 0.002778146 | 0.000567896 | 0.001372749 | 0.00024841 | 21.60044464 | 8.084373934 | 1.252986332 | 2.153883565 | 3 |
| 0.002727808 | 0.00054858 | 0.001372496 | 0.000244041 | 21.56755097 | 8.054651451 | 1.253582649 | 2.154934946 | 3 |
| 0.00269029 | 0.000531813 | 0.001374389 | 0.000245804 | 21.54671345 | 8.046638735 | 1.253112374 | 2.154559816 | 3 |
| 0.002651398 | 0.000519206 | 0.001372841 | 0.00024397 | 21.52565049 | 8.024474021 | 1.253143909 | 2.153585009 | 3 |
| 0.000328938 | 0.000115275 | 0.001163082 | 0.000144724 | 20.549147 | 6.433222532 | 1.246669226 | 1.76522601 | 4 |
| 0.00033274 | 8.70E-05 | 0.00117197 | 0.000133172 | 20.519147 | 6.433222532 | 1.248669226 | 1.79122601 | 4 |
| 0.000343074 | 0.000107854 | 0.001150911 | 0.000131573 | 20.529147 | 6.403222532 | 1.249669226 | 1.79522601 | 4 |
| 0.000278864 | 9.10E-05 | 0.001157322 | 0.000151007 | 20.539147 | 6.423222532 | 1.245669226 | 1.77522601 | 4 |
| 0.000328938 | 0.000115275 | 0.001163082 | 0.000144724 | 20.549147 | 6.433222532 | 1.246669226 | 1.76522601 | 4 |
| 0.00033274 | 8.70E-05 | 0.00117197 | 0.000133172 | 20.519147 | 6.433222532 | 1.248669226 | 1.79122601 | 4 |
| 0.000343074 | 0.000107854 | 0.001150911 | 0.000131573 | 20.529147 | 6.403222532 | 1.249669226 | 1.79522601 | 4 |
| 0.000278864 | 9.10E-05 | 0.001157322 | 0.000151007 | 20.539147 | 6.423222532 | 1.245669226 | 1.77522601 | 4 |
| 0.002877912 | -0.00315893 | -0.001249585 | 5.86E-05 | 20.549147 | 6.433222532 | 1.246669226 | 1.76522601 | 4 |
| 0.00287539 | -0.003179034 | -0.001234723 | 4.73E-05 | 20.519147 | 6.433222532 | 1.248669226 | 1.79122601 | 4 |
| 0.000343074 | 0.000107854 | 0.001150911 | 0.000131573 | 20.529147 | 6.403222532 | 1.249669226 | 1.79522601 | 4 |
| 0.000328938 | 0.000115275 | 0.001163082 | 0.000144724 | 20.549147 | 6.433222532 | 1.246669226 | 1.76522601 | 4 |
| 0.00033274 | 8.70E-05 | 0.00117197 | 0.000133172 | 20.519147 | 6.433222532 | 1.248669226 | 1.79122601 | 4 |
| 0.000343074 | 0.000107854 | 0.001150911 | 0.000131573 | 20.529147 | 6.403222532 | 1.249669226 | 1.79522601 | 4 |
| 0.000328938 | 0.000115275 | 0.001163082 | 0.000144724 | 20.549147 | 6.433222532 | 1.246669226 | 1.76522601 | 4 |
| 0.00033274 | 8.70E-05 | 0.00117197 | 0.000133172 | 20.519147 | 6.433222532 | 1.248669226 | 1.79122601 | 4 |
| 0.000343074 | 0.000107854 | 0.001150911 | 0.000131573 | 20.529147 | 6.403222532 | 1.249669226 | 1.79522601 | 4 |
| 0.000278864 | 9.10E-05 | 0.001157322 | 0.000151007 | 20.539147 | 6.423222532 | 1.245669226 | 1.77522601 | 4 |
| 0.000328938 | 0.000115275 | 0.001163082 | 0.000144724 | 20.549147 | 6.433222532 | 1.246669226 | 1.76522601 | 4 |
| 0.00033274 | 8.70E-05 | 0.00117197 | 0.000133172 | 20.519147 | 6.433222532 | 1.248669226 | 1.79122601 | 4 |
| 0.000343074 | 0.000107854 | 0.001150911 | 0.000131573 | 20.529147 | 6.403222532 | 1.249669226 | 1.79522601 | 4 |
| 0.000278864 | 9.10E-05 | 0.001157322 | 0.000151007 | 20.539147 | 6.423222532 | 1.245669226 | 1.77522601 | 4 |
| -0.00367443 | 0.002679078 | -0.001733455 | 0.000219551 | 23 | 0 | 1 | 0 | 5 |
| -0.01170152 | -0.002756834 | -7.83E-06 | 0.000356149 | 16 | 0 | 1 | 0 | 5 |
| -0.01284625 | -0.003532511 | 0.00023801 | 0.000376227 | 15 | 0 | 1 | 0 | 5 |
| -0.00482177 | 0.001902452 | -0.001486621 | 0.000238627 | 22 | 0 | 1 | 0 | 5 |
| -0.00596912 | 0.001125707 | -0.001239848 | 0.000257851 | 21 | 0 | 1 | 0 | 5 |
| -0.00826333 | -0.000427801 | -0.000746591 | 0.000296742 | 19 | 0 | 1 | 0 | 5 |
| -0.01055604 | -0.001980766 | -0.0002539 | 0.000336207 | 17 | 0 | 1 | 0 | 5 |
| 0.000912434 | 0.005782697 | -0.002720857 | 0.000144789 | 27 | 0 | 1 | 0 | 5 |
| -0.00023365 | 0.005007394 | -0.00247405 | 0.000163245 | 26 | 0 | 1 | 0 | 5 |
| -0.00138024 | 0.004231634 | -0.002227196 | 0.000181859 | 25 | 0 | 1 | 0 | 5 |
| -0.00252721 | 0.0034555 | -0.001980322 | 0.000200628 | 24 | 0 | 1 | 0 | 5 |
| -0.00367443 | 0.002679078 | -0.001733455 | 0.000219551 | 23 | 0 | 1 | 0 | 5 |
| -0.01170152 | -0.002756834 | -7.83E-06 | 0.000356149 | 16 | 0 | 1 | 0 | 5 |
| -0.01284625 | -0.003532511 | 0.00023801 | 0.000376227 | 15 | 0 | 1 | 0 | 5 |
| -0.00482177 | 0.001902452 | -0.001486621 | 0.000238627 | 22 | 0 | 1 | 0 | 5 |
| -0.00596912 | 0.001125707 | -0.001239848 | 0.000257851 | 21 | 0 | 1 | 0 | 5 |
| -0.00826333 | -0.000427801 | -0.000746591 | 0.000296742 | 19 | 0 | 1 | 0 | 5 |
| -0.01055604 | -0.001980766 | -0.0002539 | 0.000336207 | 17 | 0 | 1 | 0 | 5 |
| 0.000912434 | 0.005782697 | -0.002720857 | 0.000144789 | 27 | 0 | 1 | 0 | 5 |
| -0.00023365 | 0.005007394 | -0.00247405 | 0.000163245 | 26 | 0 | 1 | 0 | 5 |
| -0.00138024 | 0.004231634 | -0.002227196 | 0.000181859 | 25 | 0 | 1 | 0 | 5 |
| -0.00252721 | 0.0034555 | -0.001980322 | 0.000200628 | 24 | 0 | 1 | 0 | 5 |
| -0.00367443 | 0.002679078 | -0.001733455 | 0.000219551 | 23 | 0 | 1 | 0 | 5 |
| -0.01170152 | -0.002756834 | -7.83E-06 | 0.000356149 | 16 | 0 | 1 | 0 | 5 |
| -0.01284625 | -0.003532511 | 0.00023801 | 0.000376227 | 15 | 0 | 1 | 0 | 5 |
| -0.00482177 | 0.001902452 | -0.001486621 | 0.000238627 | 22 | 0 | 1 | 0 | 5 |
| -0.00596912 | 0.001125707 | -0.001239848 | 0.000257851 | 21 | 0 | 1 | 0 | 5 |
| -0.00826333 | -0.000427801 | -0.000746591 | 0.000296742 | 19 | 0 | 1 | 0 | 5 |
| -0.01055604 | -0.001980766 | -0.0002539 | 0.000336207 | 17 | 0 | 1 | 0 | 5 |
| -0.00367443 | 0.002679078 | -0.001733455 | 0.000219551 | 23 | 0 | 1 | 0 | 5 |
| -0.01170152 | -0.002756834 | -7.83E-06 | 0.000356149 | 16 | 0 | 1 | 0 | 5 |
| -0.01284625 | -0.003532511 | 0.00023801 | 0.000376227 | 15 | 0 | 1 | 0 | 5 |
| -0.00482177 | 0.001902452 | -0.001486621 | 0.000238627 | 22 | 0 | 1 | 0 | 5 |
| -0.00596912 | 0.001125707 | -0.001239848 | 0.000257851 | 21 | 0 | 1 | 0 | 5 |
| -0.00826333 | -0.000427801 | -0.000746591 | 0.000296742 | 19 | 0 | 1 | 0 | 5 |
| -0.01055604 | -0.001980766 | -0.0002539 | 0.000336207 | 17 | 0 | 1 | 0 | 5 |
| -0.00138024 | 0.004231634 | -0.002227196 | 0.000181859 | 25 | 0 | 1 | 0 | 5 |
| -0.00252721 | 0.0034555 | -0.001980322 | 0.000200628 | 24 | 0 | 1 | 0 | 5 |
| -0.00367443 | 0.002679078 | -0.001733455 | 0.000219551 | 23 | 0 | 1 | 0 | 5 |
| -0.01170152 | -0.002756834 | -7.83E-06 | 0.000356149 | 16 | 0 | 1 | 0 | 5 |
| -0.01284625 | -0.003532511 | 0.00023801 | 0.000376227 | 15 | 0 | 1 | 0 | 5 |
| -0.00482177 | 0.001902452 | -0.001486621 | 0.000238627 | 22 | 0 | 1 | 0 | 5 |
| -0.00596912 | 0.001125707 | -0.001239848 | 0.000257851 | 21 | 0 | 1 | 0 | 5 |
| -0.00826333 | -0.000427801 | -0.000746591 | 0.000296742 | 19 | 0 | 1 | 0 | 5 |
| -0.01055604 | -0.001980766 | -0.0002539 | 0.000336207 | 17 | 0 | 1 | 0 | 5 |
| 0.000912434 | 0.005782697 | -0.002720857 | 0.000144789 | 27 | 0 | 1 | 0 | 5 |
| -0.00023365 | 0.005007394 | -0.00247405 | 0.000163245 | 26 | 0 | 1 | 0 | 5 |
| -0.00138024 | 0.004231634 | -0.002227196 | 0.000181859 | 25 | 0 | 1 | 0 | 5 |
| -0.00252721 | 0.0034555 | -0.001980322 | 0.000200628 | 24 | 0 | 1 | 0 | 5 |
| -0.00367443 | 0.002679078 | -0.001733455 | 0.000219551 | 23 | 0 | 1 | 0 | 5 |
| -0.01170152 | -0.002756834 | -7.83E-06 | 0.000356149 | 16 | 0 | 1 | 0 | 5 |
| -0.01284625 | -0.003532511 | 0.00023801 | 0.000376227 | 15 | 0 | 1 | 0 | 5 |
| -0.00482177 | 0.001902452 | -0.001486621 | 0.000238627 | 22 | 0 | 1 | 0 | 5 |
| -0.00596912 | 0.001125707 | -0.001239848 | 0.000257851 | 21 | 0 | 1 | 0 | 5 |
| -0.00826333 | -0.000427801 | -0.000746591 | 0.000296742 | 19 | 0 | 1 | 0 | 5 |
| -0.01055604 | -0.001980766 | -0.0002539 | 0.000336207 | 17 | 0 | 1 | 0 | 5 |
| 0.000912434 | 0.005782697 | -0.002720857 | 0.000144789 | 27 | 0 | 1 | 0 | 5 |
| -0.00023365 | 0.005007394 | -0.00247405 | 0.000163245 | 26 | 0 | 1 | 0 | 5 |
| -0.00138024 | 0.004231634 | -0.002227196 | 0.000181859 | 25 | 0 | 1 | 0 | 5 |
| -0.00252721 | 0.0034555 | -0.001980322 | 0.000200628 | 24 | 0 | 1 | 0 | 5 |
| -0.00076048 | 0.000526861 | 0.000665348 | 3.77E-05 | 20.76083207 | 4.868898869 | 1.227512935 | 1.86104909 | 6 |
| -0.00075293 | 0.00054419 | 0.000672718 | 4.04E-05 | 20.75083207 | 4.878898869 | 1.237512935 | 1.85104909 | 6 |
| -0.00077295 | 0.000546054 | 0.00066211 | 3.68E-05 | 20.77083207 | 4.848898869 | 1.207512935 | 1.88104909 | 6 |
| -0.00077394 | 0.000535696 | 0.00066165 | 4.00E-05 | 20.77183207 | 4.858898869 | 1.217512935 | 1.87104909 | 6 |
| -0.00076048 | 0.000526861 | 0.000665348 | 3.77E-05 | 20.76083207 | 4.868898869 | 1.227512935 | 1.86104909 | 6 |
| -0.00075293 | 0.00054419 | 0.000672718 | 4.04E-05 | 20.75083207 | 4.878898869 | 1.237512935 | 1.85104909 | 6 |
| -0.00077295 | 0.000546054 | 0.00066211 | 3.68E-05 | 20.77083207 | 4.848898869 | 1.207512935 | 1.88104909 | 6 |
| -0.00077394 | 0.000535696 | 0.00066165 | 4.00E-05 | 20.77183207 | 4.858898869 | 1.217512935 | 1.87104909 | 6 |
| -0.00076048 | 0.000526861 | 0.000665348 | 3.77E-05 | 20.76083207 | 4.868898869 | 1.227512935 | 1.86104909 | 6 |
| -0.00075293 | 0.00054419 | 0.000672718 | 4.04E-05 | 20.75083207 | 4.878898869 | 1.237512935 | 1.85104909 | 6 |
| -0.00077295 | 0.000546054 | 0.00066211 | 3.68E-05 | 20.77083207 | 4.848898869 | 1.207512935 | 1.88104909 | 6 |
| -0.00076048 | 0.000526861 | 0.000665348 | 3.77E-05 | 20.76083207 | 4.868898869 | 1.227512935 | 1.86104909 | 6 |
| -0.00075293 | 0.00054419 | 0.000672718 | 4.04E-05 | 20.75083207 | 4.878898869 | 1.237512935 | 1.85104909 | 6 |
| -0.00077295 | 0.000546054 | 0.00066211 | 3.68E-05 | 20.77083207 | 4.848898869 | 1.207512935 | 1.88104909 | 6 |
| -0.00077394 | 0.000535696 | 0.00066165 | 4.00E-05 | 20.77183207 | 4.858898869 | 1.217512935 | 1.87104909 | 6 |
| -0.00076048 | 0.000526861 | 0.000665348 | 3.77E-05 | 20.76083207 | 4.868898869 | 1.227512935 | 1.86104909 | 6 |
| -0.00075293 | 0.00054419 | 0.000672718 | 4.04E-05 | 20.75083207 | 4.878898869 | 1.237512935 | 1.85104909 | 6 |
| -0.00077295 | 0.000546054 | 0.00066211 | 3.68E-05 | 20.77083207 | 4.848898869 | 1.207512935 | 1.88104909 | 6 |
| 0.001280452 | -0.002102719 | -0.001282201 | -2.96E-05 | 20.77183207 | 4.858898869 | 1.217512935 | 1.87104909 | 6 |
| 0.001297413 | -0.002116066 | -0.001281831 | -3.20E-05 | 20.76083207 | 4.868898869 | 1.227512935 | 1.86104909 | 6 |
| 0.00132042 | -0.002118582 | -0.00128908 | -2.98E-05 | 20.75083207 | 4.878898869 | 1.237512935 | 1.85104909 | 6 |
| 0.001277931 | -0.002087851 | -0.001278415 | -3.26E-05 | 20.77083207 | 4.848898869 | 1.207512935 | 1.88104909 | 6 |
| 0.001280452 | -0.002102719 | -0.001282201 | -2.96E-05 | 20.77183207 | 4.858898869 | 1.217512935 | 1.87104909 | 6 |
| -0.00076048 | 0.000526861 | 0.000665348 | 3.77E-05 | 20.76083207 | 4.868898869 | 1.227512935 | 1.86104909 | 6 |
| -0.00075293 | 0.00054419 | 0.000672718 | 4.04E-05 | 20.75083207 | 4.878898869 | 1.237512935 | 1.85104909 | 6 |
| -0.00077295 | 0.000546054 | 0.00066211 | 3.68E-05 | 20.77083207 | 4.848898869 | 1.207512935 | 1.88104909 | 6 |
| -0.00077394 | 0.000535696 | 0.00066165 | 4.00E-05 | 20.77183207 | 4.858898869 | 1.217512935 | 1.87104909 | 6 |
| -0.00076048 | 0.000526861 | 0.000665348 | 3.77E-05 | 20.76083207 | 4.868898869 | 1.227512935 | 1.86104909 | 6 |
| -0.00075293 | 0.00054419 | 0.000672718 | 4.04E-05 | 20.75083207 | 4.878898869 | 1.237512935 | 1.85104909 | 6 |
| -0.00077295 | 0.000546054 | 0.00066211 | 3.68E-05 | 20.77083207 | 4.848898869 | 1.207512935 | 1.88104909 | 6 |
| 0.002138924 | 0.002507623 | -0.000891152 | -0.000475877 | 23.42387636 | 2.836375007 | 1.227769115 | 2.100923916 | 7 |
| 0.001832951 | 0.002968923 | -0.000561354 | -0.000456844 | 23.43668698 | 2.870568396 | 1.224540156 | 2.128938226 | 7 |
| 0.004229234 | 0.001689733 | 0.000301439 | 9.47E-05 | 23.44808867 | 6.910004508 | 1.229442624 | 2.100439446 | 7 |
| 0.003862864 | 0.002120948 | 0.000663378 | 0.000113061 | 23.46729157 | 6.955796289 | 1.234709535 | 2.046027196 | 7 |
| 0.003767796 | 0.002112387 | 0.000646521 | 8.63E-05 | 23.42353976 | 6.830225382 | 1.238308459 | 2.057223094 | 7 |
| 0.003841697 | 0.002096441 | 0.00071419 | 0.00012625 | 23.53011313 | 7.037990773 | 1.244247971 | 1.950318166 | 7 |
| 0.006219139 | 0.002126053 | 0.000322026 | 0.00060711 | 23.46016279 | 8.942313147 | 1.228318323 | 2.079183044 | 7 |
| 0.00440731 | 0.00273702 | 0.000297215 | 0.000244616 | 23.45621493 | 6.860355058 | 1.234449527 | 2.084314962 | 7 |
| 0.0038127 | 0.002103251 | 0.000650464 | 9.20E-05 | 23.44852759 | 6.877212825 | 1.237662729 | 2.086216269 | 7 |
| 0.002138924 | 0.002507623 | -0.000891152 | -0.000475877 | 23.42387636 | 2.836375007 | 1.227769115 | 2.100923916 | 7 |
| 0.001832951 | 0.002968923 | -0.000561354 | -0.000456844 | 23.43668698 | 2.870568396 | 1.224540156 | 2.128938226 | 7 |
| 0.004229234 | 0.001689733 | 0.000301439 | 9.47E-05 | 23.44808867 | 6.910004508 | 1.229442624 | 2.100439446 | 7 |
| 0.003862864 | 0.002120948 | 0.000663378 | 0.000113061 | 23.46729157 | 6.955796289 | 1.234709535 | 2.046027196 | 7 |
| 0.003767796 | 0.002112387 | 0.000646521 | 8.63E-05 | 23.42353976 | 6.830225382 | 1.238308459 | 2.057223094 | 7 |
| 0.003841697 | 0.002096441 | 0.00071419 | 0.00012625 | 23.53011313 | 7.037990773 | 1.244247971 | 1.950318166 | 7 |
| 0.006219139 | 0.002126053 | 0.000322026 | 0.00060711 | 23.46016279 | 8.942313147 | 1.228318323 | 2.079183044 | 7 |
| 0.00440731 | 0.00273702 | 0.000297215 | 0.000244616 | 23.45621493 | 6.860355058 | 1.234449527 | 2.084314962 | 7 |
| 0.0038127 | 0.002103251 | 0.000650464 | 9.20E-05 | 23.44852759 | 6.877212825 | 1.237662729 | 2.086216269 | 7 |
| 0.002138924 | 0.002507623 | -0.000891152 | -0.000475877 | 23.42387636 | 2.836375007 | 1.227769115 | 2.100923916 | 7 |
| 0.001832951 | 0.002968923 | -0.000561354 | -0.000456844 | 23.43668698 | 2.870568396 | 1.224540156 | 2.128938226 | 7 |
| 0.002138924 | 0.002507623 | -0.000891152 | -0.000475877 | 23.42387636 | 2.836375007 | 1.227769115 | 2.100923916 | 7 |
| 0.006219139 | 0.002126053 | 0.000322026 | 0.00060711 | 23.46016279 | 8.942313147 | 1.228318323 | 2.079183044 | 7 |
| 0.00440731 | 0.00273702 | 0.000297215 | 0.000244616 | 23.45621493 | 6.860355058 | 1.234449527 | 2.084314962 | 7 |
| 0.0038127 | 0.002103251 | 0.000650464 | 9.20E-05 | 23.44852759 | 6.877212825 | 1.237662729 | 2.086216269 | 7 |
| 0.002138924 | 0.002507623 | -0.000891152 | -0.000475877 | 23.42387636 | 2.836375007 | 1.227769115 | 2.100923916 | 7 |
| 0.006574689 | -0.001364873 | -0.001904661 | 2.17E-05 | 23.46729157 | 6.955796289 | 1.234709535 | 2.046027196 | 7 |
| 0.006437578 | -0.001319284 | -0.001881617 | -3.62E-06 | 23.42353976 | 6.830225382 | 1.238308459 | 2.057223094 | 7 |
| 0.006602115 | -0.001451846 | -0.001899894 | 3.33E-05 | 23.53011313 | 7.037990773 | 1.244247971 | 1.950318166 | 7 |
| 0.009197269 | -0.001705556 | -0.002500696 | 0.000507015 | 23.46016279 | 8.942313147 | 1.228318323 | 2.079183044 | 7 |
| 0.007077338 | -0.000695468 | -0.002231266 | 0.000154648 | 23.45621493 | 6.860355058 | 1.234449527 | 2.084314962 | 7 |
| 0.006491754 | -0.001340389 | -0.001886498 | 1.76E-06 | 23.44852759 | 6.877212825 | 1.237662729 | 2.086216269 | 7 |
| 0.002138924 | 0.002507623 | -0.000891152 | -0.000475877 | 23.42387636 | 2.836375007 | 1.227769115 | 2.100923916 | 7 |
| 0.001832951 | 0.002968923 | -0.000561354 | -0.000456844 | 23.43668698 | 2.870568396 | 1.224540156 | 2.128938226 | 7 |
| 0.004229234 | 0.001689733 | 0.000301439 | 9.47E-05 | 23.44808867 | 6.910004508 | 1.229442624 | 2.100439446 | 7 |
| 0.003862864 | 0.002120948 | 0.000663378 | 0.000113061 | 23.46729157 | 6.955796289 | 1.234709535 | 2.046027196 | 7 |
| 0.003767796 | 0.002112387 | 0.000646521 | 8.63E-05 | 23.42353976 | 6.830225382 | 1.238308459 | 2.057223094 | 7 |
| 0.003841697 | 0.002096441 | 0.00071419 | 0.00012625 | 23.53011313 | 7.037990773 | 1.244247971 | 1.950318166 | 7 |
| 0.006219139 | 0.002126053 | 0.000322026 | 0.00060711 | 23.46016279 | 8.942313147 | 1.228318323 | 2.079183044 | 7 |
| 0.00440731 | 0.00273702 | 0.000297215 | 0.000244616 | 23.45621493 | 6.860355058 | 1.234449527 | 2.084314962 | 7 |
| 0.0038127 | 0.002103251 | 0.000650464 | 9.20E-05 | 23.44852759 | 6.877212825 | 1.237662729 | 2.086216269 | 7 |
| 0.003895924 | 0.001433145 | 0.001433648 | 0.000261502 | 23.67434242 | 8.436100543 | 1.354141827 | 1.328957105 | 7 |
| -0.00307825 | -8.83E-05 | -9.71E-05 | -0.000979704 | 22.05520735 | 0.912344378 | 1.513366717 | 3.808849927 | 7 |
| -0.00418411 | 0.000195489 | 0.000400819 | -0.000654333 | 21.24947528 | 1.34102009 | 1.49581595 | 1.746327149 | 7 |
| 0.003667406 | -0.000146958 | 0.002642861 | 0.000633264 | 22.88454419 | 11.12165909 | 1.461807715 | 0.898016762 | 7 |
| -0.00240553 | 0.000203158 | 0.000257271 | -0.000417391 | 19.7263702 | 2.656864052 | 1.306941085 | 2.151232597 | 8 |
| -0.00233601 | 0.000407007 | 0.000169133 | -0.000507031 | 19.60524648 | 2.306382443 | 1.231092924 | 1.931883499 | 8 |
| -0.0016036 | -0.000106499 | 0.000764086 | -0.000387543 | 19.45324847 | 3.862686971 | 1.215758196 | 2.139652291 | 8 |
| -0.00161198 | 0.000255052 | 0.000512908 | -0.000390337 | 19.529147 | 3.403222532 | 1.249669226 | 1.79522601 | 8 |
| -0.00055995 | 0.001194884 | -5.98E-05 | -0.000637003 | 19.79104248 | 2.420443545 | 1.243096396 | 2.041491935 | 8 |
| -0.00212344 | 0.000330963 | 0.000226431 | -0.000349335 | 19.17083207 | 2.848898869 | 1.207512935 | 1.88104909 | 8 |
| -0.00213416 | -0.000431508 | 0.000414745 | -0.000346992 | 19.57511731 | 3.433886858 | 1.249204802 | 2.8676034 | 8 |
| -0.00240553 | 0.000203158 | 0.000257271 | -0.000417391 | 19.7263702 | 2.656864052 | 1.306941085 | 2.151232597 | 8 |
| -0.00233601 | 0.000407007 | 0.000169133 | -0.000507031 | 19.60524648 | 2.306382443 | 1.231092924 | 1.931883499 | 8 |
| -0.0016036 | -0.000106499 | 0.000764086 | -0.000387543 | 19.45324847 | 3.862686971 | 1.215758196 | 2.139652291 | 8 |
| -0.00161198 | 0.000255052 | 0.000512908 | -0.000390337 | 19.529147 | 3.403222532 | 1.249669226 | 1.79522601 | 8 |
| -0.00055995 | 0.001194884 | -5.98E-05 | -0.000637003 | 19.79104248 | 2.420443545 | 1.243096396 | 2.041491935 | 8 |
| -0.00088021 | -0.001265418 | -0.000949608 | -0.000391561 | 19.17083207 | 2.848898869 | 1.207512935 | 1.88104909 | 8 |
| -0.00084586 | -0.00208573 | -0.000804009 | -0.000390683 | 19.57511731 | 3.433886858 | 1.249204802 | 2.8676034 | 8 |
| -0.00055995 | 0.001194884 | -5.98E-05 | -0.000637003 | 19.79104248 | 2.420443545 | 1.243096396 | 2.041491935 | 8 |
| -0.00212344 | 0.000330963 | 0.000226431 | -0.000349335 | 19.17083207 | 2.848898869 | 1.207512935 | 1.88104909 | 8 |
| -0.00213416 | -0.000431508 | 0.000414745 | -0.000346992 | 19.57511731 | 3.433886858 | 1.249204802 | 2.8676034 | 8 |
| -0.00240553 | 0.000203158 | 0.000257271 | -0.000417391 | 19.7263702 | 2.656864052 | 1.306941085 | 2.151232597 | 8 |
| -0.00233601 | 0.000407007 | 0.000169133 | -0.000507031 | 19.60524648 | 2.306382443 | 1.231092924 | 1.931883499 | 8 |
| -0.0016036 | -0.000106499 | 0.000764086 | -0.000387543 | 19.45324847 | 3.862686971 | 1.215758196 | 2.139652291 | 8 |
| -0.00161198 | 0.000255052 | 0.000512908 | -0.000390337 | 19.529147 | 3.403222532 | 1.249669226 | 1.79522601 | 8 |
| -0.00055995 | 0.001194884 | -5.98E-05 | -0.000637003 | 19.79104248 | 2.420443545 | 1.243096396 | 2.041491935 | 8 |
| -0.00212344 | 0.000330963 | 0.000226431 | -0.000349335 | 19.17083207 | 2.848898869 | 1.207512935 | 1.88104909 | 8 |
| -0.00213416 | -0.000431508 | 0.000414745 | -0.000346992 | 19.57511731 | 3.433886858 | 1.249204802 | 2.8676034 | 8 |
| -0.00240553 | 0.000203158 | 0.000257271 | -0.000417391 | 19.7263702 | 2.656864052 | 1.306941085 | 2.151232597 | 8 |
| -0.00233601 | 0.000407007 | 0.000169133 | -0.000507031 | 19.60524648 | 2.306382443 | 1.231092924 | 1.931883499 | 8 |
| -0.0016036 | -0.000106499 | 0.000764086 | -0.000387543 | 19.45324847 | 3.862686971 | 1.215758196 | 2.139652291 | 8 |
| -0.00161198 | 0.000255052 | 0.000512908 | -0.000390337 | 19.529147 | 3.403222532 | 1.249669226 | 1.79522601 | 8 |
| -0.00055995 | 0.001194884 | -5.98E-05 | -0.000637003 | 19.79104248 | 2.420443545 | 1.243096396 | 2.041491935 | 8 |
| -0.00212344 | 0.000330963 | 0.000226431 | -0.000349335 | 19.17083207 | 2.848898869 | 1.207512935 | 1.88104909 | 8 |
| -0.00213416 | -0.000431508 | 0.000414745 | -0.000346992 | 19.57511731 | 3.433886858 | 1.249204802 | 2.8676034 | 8 |
